# Mutational and Kinetic Analysis of Lesion Recognition by *Escherichia coli* Endonuclease VIII

**DOI:** 10.3390/genes8050140

**Published:** 2017-05-13

**Authors:** Olga A. Kladova, Alexandra A. Kuznetsova, Olga S. Fedorova, Nikita A. Kuznetsov

**Affiliations:** 1 Institute of Chemical Biology and Fundamental Medicine (ICBFM), Siberian Branch of Russian Academy of Sciences, Novosibirsk 630090, Russia; kladova@niboch.nsc.ru (O.A.K.); sandra-k@niboch.nsc.ru (A.A.K.); 2 Department of Natural Sciences, Novosibirsk State University, 2 Pirogova St., Novosibirsk 630090, Russia

**Keywords:** base excision repair, DNA glycosylase, conformational dynamics, stopped-flow enzyme kinetics, endonuclease VIII

## Abstract

*Escherichia coli* endonuclease VIII (Endo VIII) is a DNA glycosylase with substrate specificity for a wide range of oxidatively damaged pyrimidine bases. Endo VIII catalyzes hydrolysis of the N-glycosidic bond and β, δ-elimination of 3′- and 5′-phosphate groups of an apurinic/apyrimidinic site. Single mutants of Endo VIII L70S, L70W, Y71W, F121W, F230W, and P253W were analyzed here with the aim to elucidate the kinetic mechanism of protein conformational adjustment during damaged-nucleotide recognition and catalytic-complex formation. F121W substitution leads to a slight reduction of DNA binding and catalytic activity. F230W substitution slows the rate of the δ-elimination reaction indicating that interaction of Phe230 with a 5′-phosphate group proceeds in the latest catalytic step. P253W Endo VIII has the same activity as the wild type (WT) enzyme. Y71W substitution slightly reduces the catalytic activity due to the effect on the later steps of catalytic-complex formation. Both L70S and L70W substitutions significantly decrease the catalytic activity, indicating that Leu70 plays an important role in the course of enzyme-DNA catalytic complex formation. Our data suggest that Leu70 forms contacts with DNA earlier than Tyr71 does. Therefore, most likely, Leu70 plays the role of a DNA lesion “sensor”, which is used by Endo VIII for recognition of a DNA damage site.

## 1. Introduction

Endonuclease VIII (Endo VIII) from *Escherichia coli* is responsible for removal of a wide range of oxidatively damaged pyrimidine bases [1,2]. Endo VIII catalyzes hydrolysis of the N-glycosidic bond (N-glycosylase activity). As a result, the modified free base and apurinic/apyrimidinic (AP)-site are formed. The subsequent cleavage of the AP-site proceeds through β, δ-elimination (AP-lyase activity) and produces a single-stranded DNA break [3,4].

**Figure 1:**
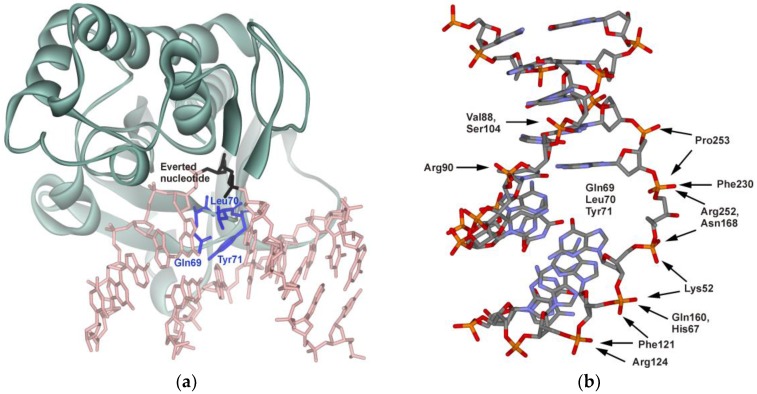
(**a**) structure of a covalently linked Endo VIII-DNA complex (Protein Data Bank (PDB) structure ID 1K3W) [6]; (**b**) functionally important amino acid residues of Endo VIII participating in formation of specific contacts between the enzyme and DNA.

Crystal structures of the free enzyme and its complexes with DNA [5,6] revealed conformational changes both in the protein and DNA. In the enzyme-DNA complex, the DNA is kinked approximately by 45° at the site of the lesion. The damaged nucleotide flips out from the helix and is inserted into the active site of the enzyme. The process of formation of the enzyme-DNA catalytic complex is accompanied by the insertion of Gln69, Leu70, and Tyr71 (numbers are given for *E. coli* protein, starting with Pro1) amino acid residues into the DNA void formed after the damage eversion (Figure 1a). Such changes in the structure of the interacting molecules lead to the formation of specific contacts, resulting in highly efficient recognition of the damaged nucleotide (Figure 1b). As shown in the figure, the enzyme interacts mainly with the damaged strand of DNA, whereas interactions between the complementary strand and enzyme are limited to a few van der Waals contacts.

**Scheme 1:**
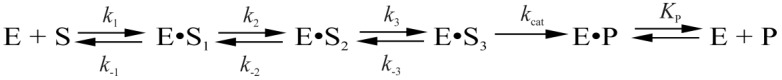
The kinetic mechanism of the interaction between Endo VIII and DNA containing a damaged base.

Protein conformational dynamics have been monitored in real time via intrinsic tryptophan (Trp) fluorescence changes [7] during interaction with DNA. Additionally, the kinetics of DNA conformational changes induced by Endo VIII have been examined using a set of fluorescent base analogs including 2-aminopurine, pyrrolocytosine, 1, 3-diaza-2-oxophenoxazine, and 3-hydroxychromone [8]. Both types of stopped-flow data [7,8] have been fitted to a kinetic scheme consisting of three reversible steps resulting in formation of an enzyme-substrate complex followed by an irreversible chemical step and equilibrium between complexed and free enzyme and DNA product (Scheme 1) [7]. It has been concluded that the first stage represents the fast initial DNA binding and formation of a nonspecific Endo VIII-DNA complex. The second stage corresponds to DNA bending, damaged-base flipping out from the double helix, and placement into the enzyme’s active site, and insertion of Gln69, Leu70, and Tyr71 into the DNA void. The following adjustment of the active site at the third stage leads to formation of a catalytically competent complex, and as a result, to the hydrolysis of the N-glycosidic bond, β-elimination of the 3′-phosphate and δ-elimination of the 5′-phosphate. The last step corresponds to dissociation of the enzyme-product complex. 

where E is Endo VIII; S is the DNA substrate; P is the DNA product of the substrate hydrolysis reaction; (E•S)_n_ are enzyme-substrate complexes; *k*_i_ and *k*_−i_ are the rate constants of direct and reverse reactions of individual stages; and *K*_P_ is the dissociation constant of the enzyme-product complex.

A similar multistep kinetic mechanism of catalytic-complex formation has been suggested for the structurally related DNA glycosylase Fpg [9,10], which is responsible for removal of oxidized purine lesions. The sequential reaction steps include: (i) formation of a nonspecific primary encounter complex between the enzyme and DNA substrate; (ii) initial lesion recognition, including destabilization of the DNA around the lesion with insertion of Phe110 into the DNA helix; (iii) formation of a kink in the DNA chain; (iv) eversion of oxoG base from the double helix into the enzyme’s active site; filling of the resulting void in the double helix by Arg108 and Met73 residues, and (v) isomerization of the enzyme to form a tight complex with the DNA substrate and to produce a catalytically active conformation.

Comparison of pro- and eukaryotic DNA glycosylases that belong to the helix-two turns-helix (H2TH) structural family revealed that these enzymes have common global structural features of DNA lesion recognition including DNA bending, damaged base flipping out of the DNA helix into the enzyme’s active site and insertion of several amino acid side chains into the void created in DNA. Therefore, the wedge strategy for the damage searching and initial recognition stages also may be common for these enzymes. In recent years, work published by Wallace and co-workers has revealed that Fpg and Endo VIII DNA glycosylases, which are members of the H2TH structural family, diffusively scan DNA using a “wedge” amino acid residue to probe for and identify oxidized nucleobases [11,12,13,14,15,16]. Moreover, some studies suggest that DNA glycosylases (Nth [14,15,17] and hOGG1 [18,19]) of another structural helix-hairpin-helix family (HhH) also use the “wedge” strategy for DNA lesion search. The function of a wedge amino acid is to sense damaged DNA bases before their eversion into the enzyme’s active site. It should be noted that, along members of H2TH structural family, there is a significant difference of the wedge organization. In Endo VIII, the intercalating/void-filling loop is formed by a continuous tripeptide (Gln69-Leu70-Tyr71 in *E. coli* Endo VIII) [6]. Replacement of a specific triad Gln69, Leu70, and Tyr71 with Ala residues (triple mutant Endo VIII Gln69Ala Leu70 Ala Tyr71 Ala) or their deletion (Δ69-71 Endo VIII) caused a dramatic decrease in DNA-binding efficiency and catalytic activity [4]. It is noteworthy that the variant of Endo VIII that contains single substitution Tyr71Ala is still enzymatically active [14], whereas, in the Fpg [20] and human NEIL1 [21], the residues come from two different loops in the protein structure (Met73, Arg108, and Phe110 in *E. coli* Fpg). Mutational analysis of Fpg revealed that Phe110 is a key “wedge” residue [10,11,22]. Indeed, the wedgeless mutant of Fpg (Phe110Ala) fails to recognize a damaged base and is devoid of catalytic activity [11,22]. At the same time, mouse NEIL3 [23] and mimivirus Nei2 [24] lack two of the three void-filling residues and are equipped with the only conserved Met residue. Continuing this line, it is clear that some void-filling amino acid residues may play a critical role in the lesion search process as a “wedge”, whereas the lack of “wedge” residues in some members of H2TH structural family required additional detail analysis of this function.

Since conformational changes of enzymes play an important role in the damage recognition and the formation of a catalytic complex, we have used detection of the intrinsic fluorescence of the enzyme’s Trp residues to study protein conformational dynamics. For the purpose of clarifying the nature of specific DNA-binding stages in the kinetic mechanism (Scheme 1), we have applied the strategy of incorporation of additional Trp residue to the different parts of the enzyme molecule to register conformational changes of these parts of enzyme. Single amino acid mutants of Endo VIII were examined using fluorescence spectroscopy and stopped-flow kinetics to identify the regions in the enzyme and DNA undergoing conformational changes and to elucidate the role of some amino acid residues (Leu70, Tyr71, Phe121, Phe230, and Pro253) in the recognition of damaged DNA.

## 2. Materials and Methods

### 2.1. Oligodeoxyribonucleotides (ODNs) and Enzymes

Mutations Leu70Ser, Leu70Trp, Tyr71Trp, Phe121Trp, Phe230Trp, or Pro253Trp within the Endo VIII coding sequence were generated using a site-directed mutagenesis kit (QuikChange XL, Stratagene, La Jolla, CA, USA). Endo VIII and its mutant forms were purified as described previously [6].

The ODNs (Table 1) were synthesized from commercially available phosphoramidites (Glen Research, Sterling, VA, USA) and purified by sequential anion exchange and reverse-phase high-performance liquid chromatography (HPLC) in the Laboratory of Biomedical Chemistry, of Institute of Chemical Biology and Fundamental Medicine of Siberian Branch of Russian Academy of Sciences (ICBFM SB RAS). The purity of ODNs exceeded 98% as estimated by electrophoresis in a 20% denaturing polyacrylamide gel after staining with the Stains-All dye (Sigma-Aldrich, St. Louis, MO, USA). The AP-containing oligonucleotide was prepared as described elsewhere [25] by incubating the deoxyuridine-containing oligonucleotide with uracil-DNA glycosylase. Reverse-phase HPLC on the Bondapak C18 column was conducted to purify the reaction product containing the AP-site [26]. Concentrations of oligonucleotides were determined by means of absorbance at 260 nm. ODN duplexes were prepared by annealing of modified and complementary strands at a 1:1 molar ratio.

### 2.2. Stopped-Flow Fluorescence Measurements

Stopped-flow measurements with fluorescence detection were essentially carried out as described elsewhere [17,27]. A model SX.18MV stopped-flow spectrometer (Applied Photophysics Ltd., Leatherhead, UK) fitted with a 150 W Xe arc lamp and 2-mm path length optical cell was used. The dead time of the instrument was 1.4 ms. Experiments were conducted in a buffer consisting of 50 mM Tris-HCl (pH 7.5), 50 mM KCl, 7% (v/v) glycerol, 1 mM dithiothreitol (DTT), 2.0 µM Endo VIII, and 0.5–4.0 µM one of DNA substrates. The temperature was 25 °C, and excitation wavelength 290 nm. The emission was monitored using a 320 nm long-pass wavelength filter (WG-320, Schott, Mainz, Germany). Each kinetic curve was averaged over at least four experimental curves.

The wild type Endo VIII contains four Trp residues: Trp26, Trp74, Trp176, and Trp256 [25]. Comparison X-ray structures of the free enzyme and its complex with the target DNA [5,6] revealed large changes in the solvent-accessible area for all these residues. This indicates that all natural Trp residues of Endo VIII may contribute to the observed fluorescence change in the course of enzyme action. Therefore, the strategy of incorporation of single Trp residue into the wild type enzyme was more preferable than additional substitution of all natural Trps with Phe, which can generate significant structural changes induced by multiple mutations.

### 2.3. Data Analysis

The processing of the fluorescence intensity traces was performed by numerical fitting of a solution of a system of ordinary differential equations corresponding to the proposed kinetic scheme using the DynaFit software (BioKin, Pullman, WA, USA) [28]. The kinetic scheme contained *n* reversible and *m* irreversible steps, and an enzyme–product complex equilibrium step and corresponding kinetic parameters were derived essentially as described previously [7,29].

### 2.4. An Assay of Relative Enzymatic Activity 

To analyze the products formed by wild-type (WT) Endo VIII and its mutant forms, ^32^P-labeled DHU- and AP-substrates were used. The solutions of the enzyme and ^32^P-labeled substrate were mixed for 30 s in a buffer consisting of 50 mМ Tris-HCl pH 7.5, 50 mМ KCl, 1 mМ ethylenediaminetetraacetic acid (EDTA), 1 mМ dithiothreitol, and 7% (v/v) of glycerol at 25 °C. The concentration of DNA substrates was 1.0 μM. The concentration of enzymes was 2.0 or 1.0 μM in the case of DHU-substrate or AP-substrate, respectively. After mixing of solutions of the enzyme and DNA substrate, a 2 μL aliquot was placed in already prepared test tubes containing 3 μL of 7 M urea, 0.1% of bromophenol blue, and 0.1% of xylene cyanol FF. Polyacrylamide gel electrophoresis (PAGE) was conducted at 50 V/cm. The gel was visualized by autoradiography using Agfa CP-BU X-ray film (Agfa-Gevaert, Mortsel, Belgium). The autoradiograms were scanned and quantified on a Gel-Pro Analyzer software (version, Media Cybernetics, Rockville, MD, USA).

## 3. Results

In this study, we aimed to gain a better insight into the process of recognition of specific DHU- and AP-substrates by Endo VIII. The amino acid residues Leu70, Tyr71, Phe121, Phe230, and Pro253, which are important for DNA binding (Figure 1), were substituted to obtain the following Endo VIII mutants: Leu70Ser, Leu70Trp, Tyr71Trp, Phe121Trp, Phe230Trp, and Pro253Trp. In most cases, specific amino acids were substituted with fluorescent Trp, which enables monitoring of conformational transitions in the vicinity of these residues.

**Figure 2:**
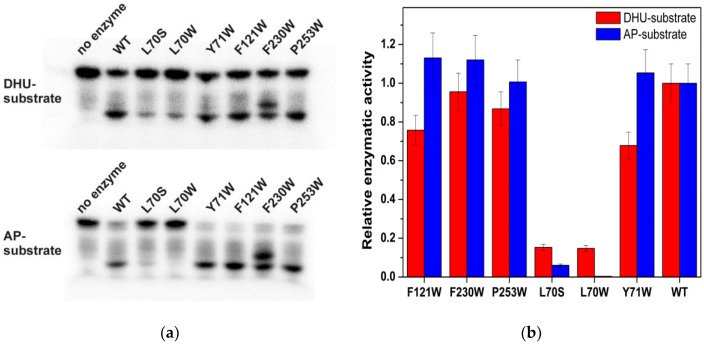
Enzymatic activity of Endo VIII and its mutant forms during interaction with the DHU-substrate and AP-substrate. (**a**) accumulation of a reaction product according to PAGE (polyacrylamide gel electrophoresis). (**b**) relative cleavage activity of Endo VIII and its mutant forms with the DHU-substrate and AP-substrate.

The relative activity of the mutant enzymes was determined by direct PAGE analysis of the accumulation of products derived from the ^32^P-labeled DHU-substrate and AP-substrate (Figure 2). As shown in Figure 2, the strongest effect of the amino acid substitutions is caused by Leu70 mutations. The mutant forms F230W, F121W, P253W, and Y71W have similar enzymatic activities, which are close to that of WT.

It should be noted that, in the case of the F230W mutant form, there are two product bands are registered. Wild type Endo VIII catalyzes two fast sequential reactions: β-elimination of the 3′-phosphate and δ-elimination of the 5′-phosphate. Therefore, the appearance of two products indicates that, in the case of F230W, the second reaction of δ-elimination of the 5′-phosphate proceeds more slowly than for WT, and bands correspond to the products of β- and δ-elimination reaction, respectively.

**Scheme 2:**
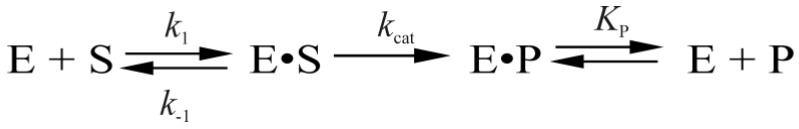
The kinetic scheme of L70W and L70S interaction with the DHU- or AP-substrate.

**Figure 3:**
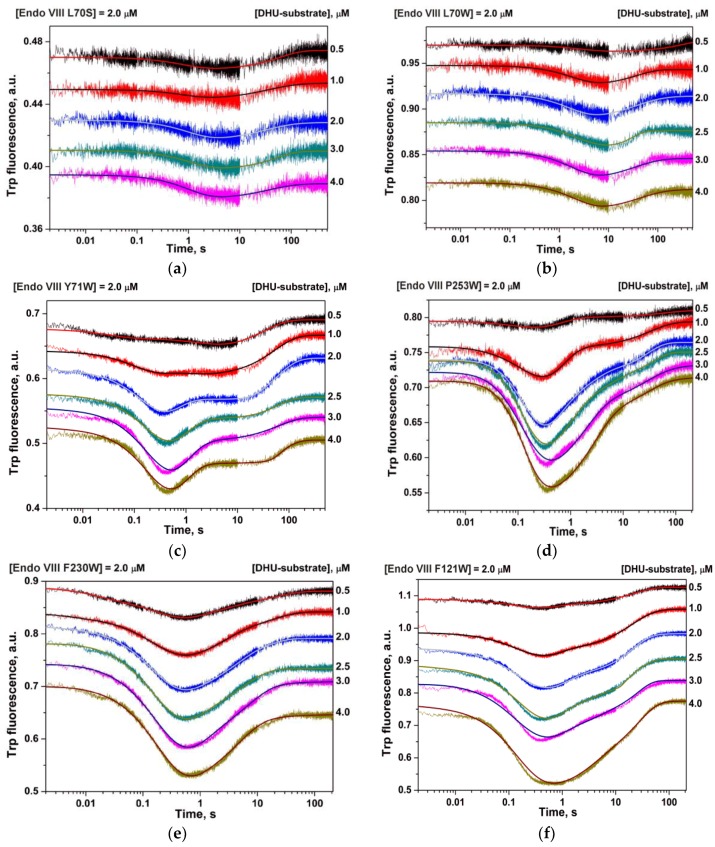
Changes in Trp fluorescence intensity during the interaction of Endo VIII mutants—(**a**) L70S; (**b**) L70W; (**c**) Y71W; (**d**) P253W; (**e**) F230W; and (**f**) F121W—with the DHU-substrate. The concentrations of enzymes and DNA substrate are indicated in the panels.

**Figure 4:**
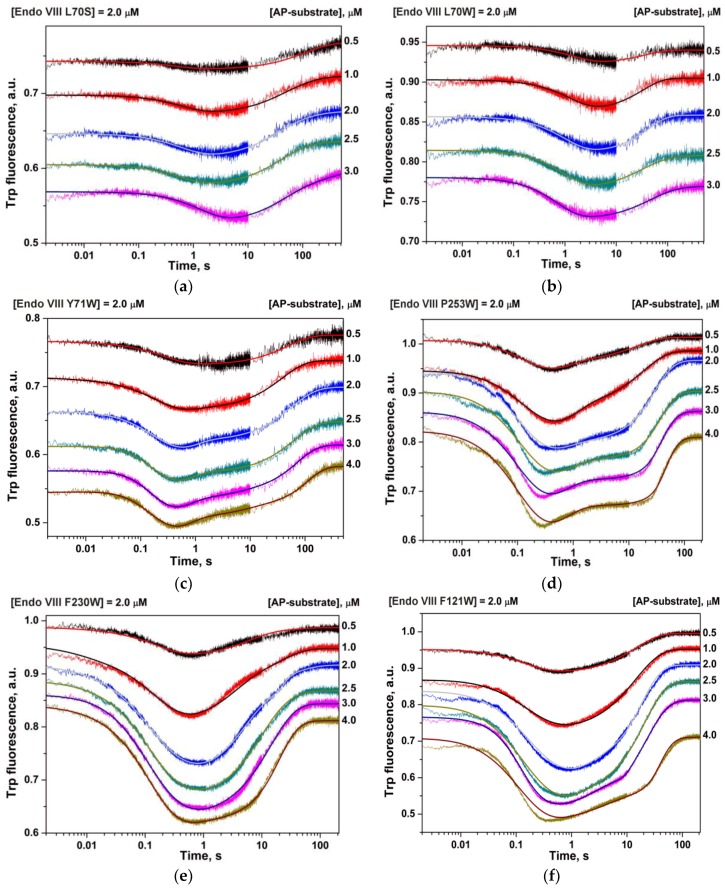
Changes in Trp fluorescence intensity during the interaction of Endo VIII mutants—(**a**) L70S; (**b**) L70W; (**c**) Y71W; (**d**) P253W; (**e**) F230W; and (**f**) F121W—with the AP-substrate. Concentrations of the enzymes and substrate are indicated in the panels.

The analysis of real-time conformational changes of the enzymes allows us to specify the role of selected amino acid residues during DNA binding and catalysis. For this purpose, pre-steady state kinetics of the enzymatic process was studied by the stopped-flow technique. The changes in intrinsic fluorescence intensity of tryptophan as a marker of conformational transitions in the enzyme were monitored during interaction of mutant forms of Endo VIII with the DHU-substrate or AP-substrate (Figure 3 and Figure 4, respectively). The experimental data obtained for Y71W, F121W, F230W, or P253W at different concentrations of a DNA substrate were acceptably fitted to Scheme 1 proposed for WT Endo VIII [7]. It includes three reversible steps corresponding to the enzyme binding to the substrate and formation of a catalytically active complex, one irreversible catalytic step, and a reversible step of product release from the complex with the enzyme. At the same time, the experimental data on L70W and L70S were fitted to Scheme 2. In contrast to kinetic Scheme 1, it contains a single equilibrium step of the enzyme’s binding to DNA, one irreversible catalytic step, and a reversible product release step. The fitted kinetic parameters obtained for the DHU-substrate and AP-substrate are listed in Table 2 and Table 3, respectively.

where E is Endo VIII, S is the DNA substrate, P is the DNA product of the substrate hydrolysis reaction, E•S is the enzyme–substrate complex; *k*_i_ and *k*_−i_ are rate constants of direct and reverse reactions of individual stages; and *K*_P_ is the dissociation constant of the enzyme–product complex.

## 4. Discussion

The set of mutant forms of Endo VIII that contain a Trp residue in various parts of the enzyme–DNA complex was analyzed to elucidate the kinetic mechanism of protein conformational adjustment during damaged-nucleotide recognition and catalytic-complex formation. According to existing data [7,8], the first step in the kinetic mechanism (Scheme 1) corresponds to the initial DNA binding and closing of domains; the second step corresponds to the DNA kinking, the damaged base flipping out and insertion of the enzyme’s loop into the void; the third stage is adjustment of the enzyme’s active site; the fourth stage corresponds to the damaged-base excision; and the fifth stage denotes the product release.

**Figure 5:**
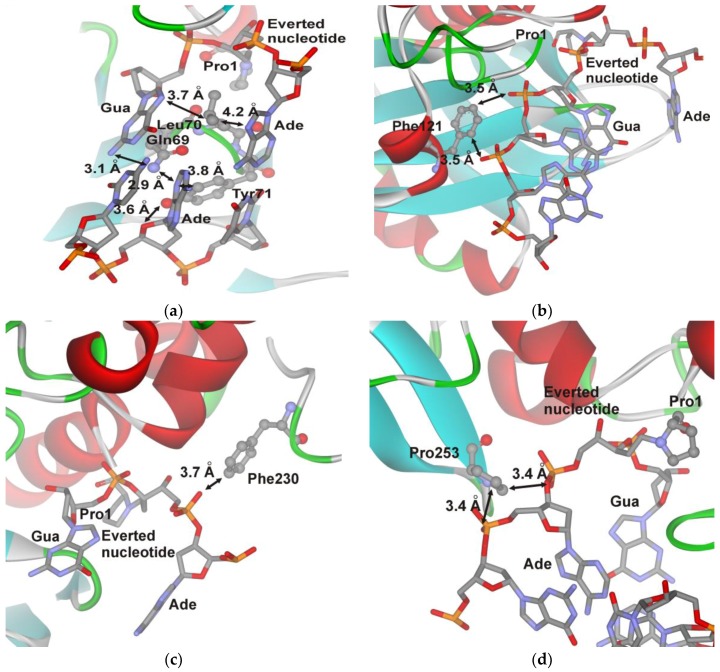
Close-up view of the important contacts in the DNA-binding cleft of the covalent complex of Endo VIII with DNA. The van der Waals interactions between DNA and the Gln69, Leu70, and Tyr71 triad (**a**); Phe121 (**b**); Phe230 (**c**); and Pro253 (**d**) are shown. The arrows indicate the distances (**a**) between Tyr71 Cε1 and N3 of the Ade base located opposite of the damaged nucleotide (3.8 Å); between Tyr71 Oη and O4’ of the nucleotide (3.6 Å); between Leu70 Cδ1 and N9 of the A and G bases (4.2 or 3.7 Å, respectively); (**b**) between Phe230 Cζ and the O atom of the phosphate group (3.7 Å); (**c**) between Phe121 Cε1 or Cζ and the O atom of phosphate groups (both 3.5 Å); (**d**) between Pro253 Cδ and the bridge O atom of the phosphate group or Pro253 Cγ and the O atom of the phosphate group (both 3.4 Å).

Leu70 and Tyr71 were selected as possible “wedge” amino acids. The X-ray structural results revealed that both Leu70 and Tyr71 have direct contacts with DNA bases located opposite to or on the 5′ and 3′ side of the damaged nucleotide (Figure 5a). Residue Phe121 is located in the peptide linker connecting the two protein domains forming the DNA-binding site and interacts with phosphate groups of DNA (Figure 5b). The Phe230 residue participates in formation of the damaged-base-binding pocket via interaction with a phosphate group (Figure 5c). Residue Pro253 is located in the enzyme’s DNA-binding site; and it interacts with the phosphate groups located on the 5′ side of the damaged nucleotide (Figure 5d). 

**Figure 6:**
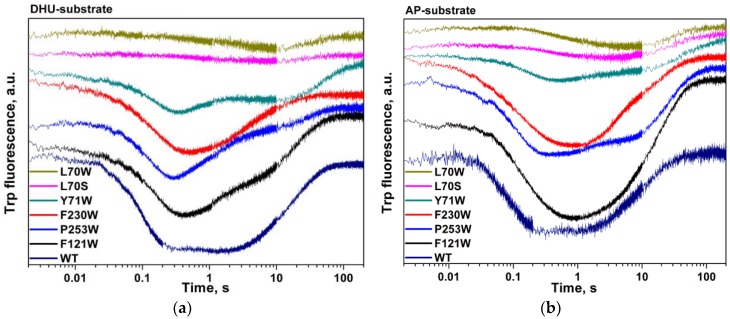
Changes in Trp fluorescence intensity during the interaction of Endo VIII and its mutants with the DHU-substrate (**a**) or AP-substrate (**b**). The concentrations of enzymes and DNA substrates were 2 µM. The kinetic traces are manually offset for clarity.

The PAGE product analysis revealed that enzyme interaction with DHU- or AP-substrate is nearly unaffected by the substitutions of Tyr71, Phe121, Phe230, and Pro253 with Trp (Figure 2). The maximal differences were observed for L70S and L70W mutants, which almost completely lost the activity on the AP-substrate and showed more than fivefold decrease in the catalytic activity on the DHU-substrate. According to Figure 6, all of the mutant forms of the enzyme have some differences in the amplitude and shape of fluorescent kinetic traces. Indeed, the reduction in the enzymatic activity induced by an amino acid substitution reduces the amplitude of the Trp fluorescence drop in the initial part of the kinetic trace. Moreover, different behaviors of the fluorescence signal in the time range up to 5 s (which characterizes steps of the specific-complex formation) revealed a movement of the new Trp residue.

In the case of F121W, the total association constant *K*_ass_ is fourfold higher than that of WT. Nevertheless, due to about a twofold lower value of *k*_cat_, this mutant is about twofold less active than WT Endo VIII is at saturating concentrations of the substrate.

Phe230 is one of the amino acids forming the active site pocket, in which the damaged base is placed. Moreover, Phe230 interacts with 5′-phosphate group of the damaged nucleotide (Figure 5c). Tolerance to the F230W substitution (Table 2 and Table 3) reveals that the Trp residue can perform similar contacts with the extruded base as Phe residue. However, substitution F230W retards the reaction of δ-elimination of the 5′-phosphate (Figure 2a) indicating that interaction of Trp230 with 5′-phosphate group proceeds in the latest step of catalytic reaction.

Meanwhile, P253W mutant has a similar activity as the WT. Pro253 residue is located in the enzyme’s DNA-binding site; therefore, a lack of effect of a Trp substitution on kinetic rate constants can indicate two possibilities: Trp retains the same contacts with DNA as Pro253 or contacts between Pro253 and the substrate’s ribose-phosphate backbone (Figure 5d) are not so important for achieving the catalytic competent state.

Structural [6] and mutational [14] data suggest that Tyr71 can serve as the “wedge” residue. Nonetheless, substitution Y71W only slightly reduced the catalytic activity. Moreover, according to [14], Endo VIII Y71A (amino acid numbers started with Pro1) retains nearly wild type catalytic activity. This data suggest that the contacts formed by Tyr71 are not very important for catalytic-complex formation. The Y71W substitution leads to a decrease in the amplitude of Trp fluorescence changes in comparison with WT in our pre-steady state kinetic traces. Analysis of the rate constant revealed that Y71W affects the second and third steps (Table 2 and Table 3) of catalytic-complex formation and overall reduces DNA-binding constant *K*_ass_ four- to tenfold as compared with the WT.

The results presented here show that substitution of residue Leu70 highly affects both catalytic and binding steps. Substitutions L70S and L70W lead to a significant (~5- to 10-fold) decrease in the catalytic activity (Figure 2) and in the amplitude of the decreasing phase of Trp fluorescence (Figure 6). The decrease in the Trp fluorescence proceeds slowly (~10 s, Figure 6a) as compared with the WT (~0.2 s, Figure 6a), indicating that residue Leu70 is important at the earliest step of enzyme–DNA complex formation. Most likely, the substitution of Leu70 perturbs the network contacts that are important for initial damage recognition.

Mutants L70W and L70S execute only one-step binding (Scheme 2), which proceeds ~300-fold more slowly than does the first binding step of other mutants and WT Endo VIII. The affinity of L70W and L70S for the DHU-substrate is tenfold lower than that in other cases (compare the values of *K*_1_ for L70W and L70S with *K*_ass_ for other mutants and the WT enzyme in Table 2), whereas their *k*_cat_ values are fourfold lower than *k*_cat_ for the WT. Comparing these data with Scheme 1, it is reasonable to conclude that the second and third binding steps leading to the active enzyme–substrate complex still take place because both mutants have some catalytic activity. These steps could not be resolved in fluorescence traces if they were limited by previous steps. Our kinetic data suggest that amino acid residue Leu70 protrudes from the enzyme globule and forms contacts with DNA earlier than Tyr71 does. Therefore, results presented here establish that the Leu70 is a DNA lesion “sensor” that is used by Endo VIII for searching the DNA damage site. Nevertheless, the Tyr71 residue is required for stabilization of the extruded conformation of the damaged base and for reorganization of the enzyme structure in a catalytically competent state.

## 5. Conclusions

In order to elucidate the role of some amino acid residues (Leu70, Tyr71, Phe121, Phe230, and Pro253) of Endo VIII in the recognition of DNA lesions we have studied the conformational dynamics of wildtype enzyme and single amino acid mutants using stopped-flow fluorometry. Taken together, our results suggest that the Leu70 acts as a DNA lesion “sensor” for searching the DNA damaged site by Endo VIII.

## Figures and Tables

**Table 1:** Sequences of oligodeoxynucleotide duplexes used in this work *.

Shorthand	Sequence
DHU-substrate	TCTCTC(DHU)CCTTCC AGAGAG G GGAAGG
AP-substrate	TCTCTC(AP)CCTTCC AGAGAG G GGAAGG
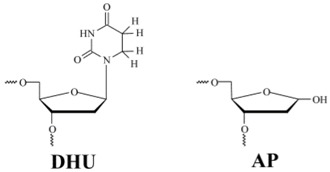	

* DHU is dihydrouracil, AP is apurinic/apyrimidinic site.

**Table 2:** The kinetic parameters of DHU-substrate cleavage by wild-type Endo VIII and its mutant forms.

Constants	L70S	L70W	Y71W	F121W	F230W	P253W	WT [7]
*k*_1_, M^−1^s^−1^	(0.09 ± 0.02) × 10^6^	(0.06 ± 0.01) × 10^6^	(21 ± 3) × 10^6^	(39 ± 11) × 10^6^	(30 ± 2) × 10^6^	(27 ± 2) × 10^6^	(36 ± 7) × 10^6^
*k*_−1_, s^−1^	1 ± 0.1	0.4 ± 0.1	330 ± 30	120 ± 31	310 ± 45	310 ± 26	410 ± 20
*K*_1_, M^−1^	0.9 × 10^5^	1.5 × 10^5^	0.63 × 10^5^	3 × 10^5^	0.97 × 10^5^	0.87 × 10^5^	0.88 × 10^5^
*k*_2_, s^−1^			16 ± 3	17 ± 4	21 ± 3	23 ± 3	27 ± 2
*k*_−2_, s^−1^			2.5 ± 0.1	0.55 ± 0.25	1.2 ± 0.1	0.8 ± 0.1	1.8 ± 0.3
*K*_2_			6.4	31	17.5	28.7	15
*k*_3_, s^−1^			0.4 ± 0.1	0.58 ± 0.15	0.82 ± 0.04	1.0 ± 0.03	1.6 ± 0.1
*k*_−__3_, s^−1^			1.1 ± 0.1	0.89 ± 0.14	0.66 ± 0.06	1.2 ± 0.1	1.5 ± 0.2
*K*_3_			0.36	0.65	1.2	0.83	1.1
*K*_ass_, M^−^^1^			1.45 × 10^5^	6 × 10^6^	2 × 10^6^	2 × 10^6^	1.45 × 10^6^
*k*_cat_, s^−^^1^	0.09 ± 0.01	0.08 ± 0.03	0.14 ± 0.03	0.26 ± 0.09	0.38 ± 0.01	0.4 ± 0.03	0.35 ± 0.02
*K*p, M	(1.0 ± 0.2) × 10^−^^6^	(0.7 ± 0.5) × 10^−^^6^	(0.4 ± 0.1) × 10^−^^6^	(0.6 ± 0.1) × 10^−^^6^	(0.6 ± 0.2) × 10^−^^6^	(0.63 ± 0.08) × 10^−^^6^	(0.7 ± 0.1) × 10^−^^6^

*K*_i_ = *k*_i_/*k*_−i_, i is the ID number of a stage, *K*_ass_ = *K*_1_*K*_2_*K*_3_.

**Table 3:** The kinetic parameters of AP-substrate cleavage by wild-type Endo VIII and its mutant forms.

Constants	L70S	L70W	Y71W	F121W	F230W	P253W	WT [7]
*k*_1_, M^−1^s^−1^	(0.09 ± 0.01) × 10^6^	(0.11 ± 0.02) × 10^6^	(14 ± 2) × 10^6^	(16 ± 1) × 10^6^	(18 ± 8) × 10^6^	(18 ± 3) × 10^6^	(12 ± 1) × 10^6^
*k*_−1_, s^−1^	0.9 ± 0.03	0.6 ± 0.05	170 ± 17	132 ± 12	81 ± 32	130 ± 28	260 ± 10
*K*_1_, M^−1^	1 × 10^5^	1.8 × 10^5^	0.8 × 10^5^	1.2 × 10^5^	2.2 × 10^5^	1.4 × 10^5^	0.5 × 10^5^
*k*_2_, s^−1^			21 ± 2	35 ± 3	25 ± 6	37 ± 4	30 ± 1
*k*_−2_, s^−1^			1.4 ± 0.1	0.9 ± 0.3	1.0 ± 0.3	1.9 ± 0.5	4.4 ± 0.2
*K*_2_			15	39	25	19	6.8
*k*_3_, s^−1^			0.13 ± 0.01	0.8 ± 0.1	0.9 ± 0.1	0.35 ± 0.02	2.1 ± 0.1
*k*_−__3_, s^−1^			1.0 ± 0.1	3.0 ± 0.6	4.7 ± 1.1	1.9 ± 0.2	1.1 ± 0.1
*K*_3_			0.13	0.27	0.19	0.18	1.9
*K*_1_*K*_2_*K*_3_, M^−^^1^	1 × 10^5^	1 × 10^5^	1.6 × 10^5^	12.6 × 10^5^	10.5 × 10^5^	4.9 × 10^5^	6.5 × 10^5^
*k*_cat_, s^−^^1^	0.09 ± 0.01	0.08 ± 0.007	0.29 ± 0.03	0.41 ± 0.05	1.0 ± 0.1	0.45 ± 0.08	0.79 ± 0.03
*K*p, M	(0.18 ± 0.02) × 10^−^^6^	(0.2 ± 0.1) × 10^−^^6^	(0.11 ± 0.01) × 10^−^^6^	(1.5 ± 0.4) × 10^−^^6^	(1.1 ± 0.6) × 10^−^^6^	(1.4 ± 0.2) × 10^−^^6^	(1.8 ± 0.3) × 10^−^^6^

*K*_i_ = *k*_i_/*k*_-i_, i is the ID number of a stage, *K*_ass_ = *K*_1_*K*_2_*K*_3_.
